# Relation of adiposity, television and screen time in offspring to their parents

**DOI:** 10.1186/1471-2431-13-133

**Published:** 2013-09-03

**Authors:** Lyn M Steffen, Alan R Sinaiko, Xia Zhou, Antoinette Moran, David R Jacobs Jr, Yoel Korenfeld, Donald R Dengel, Lisa S Chow, Julia Steinberger

**Affiliations:** 1 Division of Epidemiology and Community Health, University of Minnesota School of Public Health, 1300 South Second St Suite 300, Minneapolis, MN 55454, USA; 2 Department of Pediatrics, University of Minnesota School of Medicine, 1300 South Second St Suite 300, Minneapolis, MN 55454, USA; 3 School of Kinesiology, University of Minnesota, 1300 South Second St Suite 300, Minneapolis, MN 55454, USA; 4 Division of Endocrinology, Diabetes and Metabolism, University of Minnesota School of Medicine, 1300 South Second St Suite 300, Minneapolis, MN 55454, USA

**Keywords:** Offspring, Parents, Adiposity, Television, Total screen time

## Abstract

**Background:**

Few studies have examined the relations of adiposity and lifestyle factors in young offspring with their parents as children (parents_child_) or at their current age (parents_adult_). Therefore, we compared measures of adiposity and lifestyle in parents_child_ and parents_adult_ with their offspring.

**Methods:**

Two generations (one parent and his/her offspring) participated in this study: 234 parents from a previously established cohort and 382 offspring. Parents_adult_ and offspring underwent measurements for height, weight, waist circumference, % body fat, visceral fat, and lifestyle habits. Participants were classified as normal weight, overweight, obese based on age-specific BMI criteria. Mixed model linear regression analysis evaluated the associations of adiposity and lifestyle factors of parents_child_ and parents_adult_ with that of their offspring, adjusting for age, sex, race, and family membership.

**Results:**

The prevalence of obesity was greater among offspring mean age 12.3 years compared to their parents_child_ mean age 12.6 years (18.4% vs 10.1%, p<0.001) even though hours of television (TV) watching were similar between the two generations as children (p=0.80). Sixty percent of parents (as children and adults) and offspring reported more than 2 hours of TV/day. Offspring of parents who were overweight and obese as children had greater BMI (all p<0.001) than offspring of parents who were normal weight as children. For both parent_adult_ and offspring, adiposity was greater with greater total screen time.

**Conclusions:**

Identifying high-risk families is important for early intervention of overweight, especially in children.

## Background

It is well-known that obesity is related to increased risk of cardiovascular disease (CVD) and diabetes [1]. Parental obesity doubles the risk that their offspring will be obese adults, whether the offspring were obese or non-obese in childhood [2,3]. Therefore, children of obese parents have greater susceptibility for the development of CVD and diabetes than children of normal weight parents [4-6]. Whether these associations between parents and children can be related to the degree of parental obesity when they were children is not known. Nor is it known whether distribution of fat (i.e., abdominal, visceral, subcutaneous) in parents is related to levels of adiposity or distribution of fat in their children. Visceral adipose tissue (VAT) and subcutaneous adipose tissue (SAT), are known to increase metabolic risk [7], and prior studies of parents and their adult offspring have shown a genetic linkage for abdominal visceral fat [7-9].

Sedentary behaviors, e.g., watching television (TV) or using the computer, have been associated with overweight among children and adults [10,11], and family environment influences television viewing and physical activity behaviors [12]. Studies have shown more hours of TV watching and computer use among children of overweight and obese parents than children of normal weight parents [13,14]. Little is known about the relation of TV time or total screen time with specific measures of adiposity, including VAT and SAT, among families.

The objectives of the present study were to compare measures of adiposity and lifestyle between parents and their offspring. This study adds to previously published studies by comparing the measures of parents in adulthood to their offspring but also adding new evidence by comparing parent measures obtained when they were children to those of their offspring at similar ages. We hypothesized that levels of adiposity in the offspring would be related to the same patterns of adiposity observed in their parents during both adulthood as well as when they were children. We further hypothesized that levels of sedentary behaviors (TV watching, total screen time) would be significantly correlated between parents and their offspring and related to their levels of adiposity.

## Methods

The Institutional Review Board at the University of Minnesota approved this study. Adult study participants signed informed consent forms for themselves and their young offspring, while children signed assent forms.

The present study was conducted in a cohort of parents (N=234) who had participated in a longitudinal study, beginning at age 6–9 years [15] and continuing to age 39 [16], and their offspring (N=382) >6 years of age. Because parental participation in this study was based on their prior childhood enrollment, only one parent per child was eligible for the study. Parents with chronic disease including type 2 diabetes, end-stage kidney disease and cancer (n=14) were not eligible to participate in the present clinic examination. Parents during their childhood will be represented as parent_child_ and parents at their current age or adult will be represented by parent_adult_.

### Anthropometric measurements

Parents_adult_ and offspring were examined using standardized protocols. Standing height was measured to the nearest cm. Weight in kg was determined by a balance scale. Body mass index (BMI) was calculated as weight (kg) divided by height (m^2^). Standard BMI categories were used for parents_adult_: normal weight (BMI<25 kg/m2), overweight (BMI 25<30) and obese (BMI ≥30). For parents_child_ and offspring, standard age and gender specific childhood BMI classifications were used: normal weight (BMI<85 percentile), overweight (BMI 85–95 percentile) and obese (BMI>95 percentile) [17]. Percent (%) body fat, fat free mass and central adiposity were determined by dual-energy x-ray absorptiometry (DXA) with a Lunar Prodigy scanner (pediatric software version 9.3; General Electric Medical Systems, Madison, WI, USA), in the total body scanning mode [18]. Visceral adipose tissue (VAT) and total fat were measured by abdominal CT scan at the level of L4-L5 disk space using a Siemens Somatom Plus 4 scanner, with adipose tissue calculated by identifying pixels with attenuation values between −190 and −30 Hounsfield units (HU) [19].

### Lifestyle characteristics

For the current visit, standardized questionnaires were administered to parents_adult_ and their offspring to obtain information about lifestyle habits including sedentary and physical activities. Participants were asked about TV programs watched each day for the previous 7 days, including name of program, program length (minutes), and whether the entire program was viewed. For the 7-day TV recall, a local TV guide was used to assist study participants in recording TV programs and number of minutes watched. Questions were included about computer and video games use. Data about hours of TV watching were available for the parent_child_ at the first clinic visit when they were mean age 7 years; however, different physical activity questionnaires were administered in the previous and more recent studies. The physical activity questionnaire administered to parents_child_ queried organized and individual sports participation during the past year (response choices included never, occasionally, often); however, this questionnaire was not validated. The validated Godin Leisure Time physical activity questionnaire was administered to offspring and queried sedentary, moderate, and vigorous intensity physical activity during the previous 7 days [20]. Although both questionnaires asked about performance of moderate and vigorous physical activities, the time frame was different. Thus, we were unable to compare physical activity habits between parents_child_ and their offspring.

### Statistical analyses

Statistical analysis was performed using SAS, version 9.2 (SAS Institute; Cary, NC). Demographic and clinical characteristics of parents_adults_, their offspring, and for parents_child_ (at a similar age as their offspring) were expressed as means ± standard deviation (SD) for continuous variables and frequencies (%) for categorical variables. Pearson correlations of characteristics were determined between parents at both ages and offspring. Taking into account family membership, mixed model linear regression analysis was used to evaluate the relations of characteristics of parents_adult_ and parents_child_ with offspring, adjusted for age, sex, and race. In addition, using mixed model regression analysis the relations were examined for offspring characteristics across parent BMI categories (as children and adults). Because stratified analyses by age of the offspring (<10 yrs; >=10 yrs) yielded the same results for both age groups, results are presented for the offspring as a single cohort. Finally, multiple linear regression analyses were conducted to determine which of the parental BMI measures (as a child or adult) more strongly influenced offspring adiposity. Parental adult and/or child BMI variable were included as independent variables in the model(s) to predict the (dependent variable) offspring adiposity variable.

## Results

### Comparison between parents at their current adult age (parent_adult_) and their offspring

Demographic and clinical characteristics of the parents_adult_ age and their offspring are presented in Table 1. The proportion of non-white parents was 38.5% and 48% offspring. Pearson correlation coefficients between parents_adult_ and offspring were significant (r=0.28-0.47) for BMI, waist circumference, % body fat, VAT, and SAT. Mean total screen time and sessions of moderate and vigorous physical activity were higher among offspring compared to their parents_adult_, but there was a significant correlation between parents and their offspring for hours spent in sedentary activity, including TV watching, computer and video game time, and total screen time, but not for physical activity. Correspondingly, a similar proportion of parents and their offspring reported 2 or more hours of total screen time, including hours of watching TV.

**Table 1:** Mean ± SD, frequency (%), and correlations of characteristics of parents as adults (n=234) and their offspring (n=382)

**Characteristics**	**Parent as adult (n=234)**	**Offspring (n=382)**	**Pearson correlation**^**1**^
*Demographics*			
Age, years	39.1 ± 1.5	12.2 ± 4.6	--
Sex, Male, n (%)	144 (62)	203 (53)	--
Race, n (%)			
White	144 (61.5)	198 (52)	--
African American	65 (28)	131 (35)	
Hispanic	1 (0.5)	3 (1.0)	
Other	24 (10)	47 (12)	
Education, years	14.5 ± 2.8	n/a	--
*Clinical characteristics*			
BMI, kg/m^2^	30.3 ± 7.3	21.4 ± 6.5	0.47**
BMI status, n (%) ^2^			
Normal weight	60 (25)	248 (65)	--
Overweight	67 (29)	64 (17)	
Obese	107 (46)	70 (18)	
Waist circumference, cm	100.5 ± 18.6	73.0 ± 17.1	0.35**
% body fat	38.0 ± 10.6	25.8 ± 12.0	0.28**
Visceral adipose tissue, cm^2^	60.9 ± 32.2	18.1 ± 13.3	0.28**
Subcutaneous adipose tissue, cm^2^	176.1 ± 91.9	77.1 ± 79.1	0.43**
*Lifestyle factors*			
Current smoking, n (%)	147 (63)	55 (19)^3^	--
TV hours, hours/day	3.0 ± 2.1	2.9 ± 1.9	0.37**
> 2 hours of TV/day, n (%)	132 (57.1)	224 (60.4)	
Computer or video, hours/day***	1.4 ± 1.4	2.2 ± 2.6	0.14*
Total screen time, hours/day***	4.4 ± 2.8	5.1 ± 3.7	0.31**
> 2 hours of screen time/day, n (%)	185 (80.9)	298 (80.3)	
Physical activity sessions^4^	3.9 ± 3.9	8.7 ± 7.2	0.06

^1^ Pearson correlations for adiposity factors between parents at their current age and offspring.

^2^ For children, BMI status is represented as BMI percentiles <85, 85–95, >=95. For adults, BMI status is represented as BMI <25, 25–30, >=30 kg/m^2^.

^3^ Smoking questions only for children >10 years old.

^4^ Sum of moderate + vigorous physical activity sessions/week.

*p<=0.01; **p<0.001; ***Total screen time n=231 for parents; n=369 for offspring.

**Figure 1:**
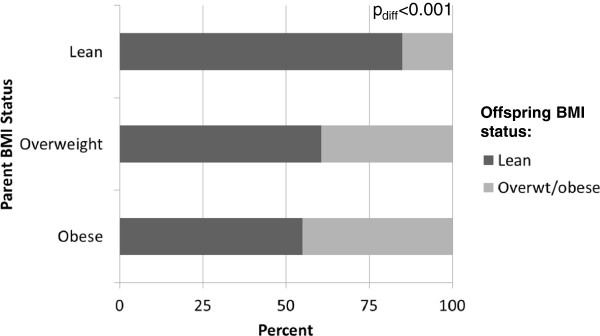
Distribution of normal weight, overweight, and obese children according to parental BMI status (as adults).

The distribution of BMI categories in offspring was significantly associated with their parents’ adult BMI status (Figure 1). A greater proportion of normal weight offspring had normal weight parents than overweight or obese parents, and a greater proportion of overweight/obese offspring had overweight or obese parents than normal weight parents (p<0.001). BMI, waist circumference, % body fat, and visceral and subcutaneous adipose tissue were significantly greater in offspring of parents who were overweight or obese than offspring of normal weight parents (Table 2).

**Table 2:** Mean±SE level or frequency (%) of measures of adiposity for offspring (n=382) according to parent BMI status adjusted for offspring’s age, sex, and race (white, non-white) (n=234)

	**Parent BMI status (current age)**			**p-value**
	**Normal weight n = 102**	**Overweight n = 108**	**Obese n = 172**	
BMI of parent at current age	21.9	27.9	36.4	<0.001
***Risk factors in Offspring***				
Age, years	11.2 ± 0.4	12.3 ± 0.4	12.9 ± 0.3	0.008
BMI, kg/m^2^	19.2 ± 0.5 ^a,b^	21.0 ± 0.5 ^a,c^	23.1 ± 0.4 ^b,c^	<0.001
BMI percentile	47.3 ± 2.7 ^a,b^	66.8 ± 2.7 ^a,c^	73.4 ± 2.2 ^b^	<0.001
Waist circumference, cm	67.9 ± 1.3 ^a,b^	72.7 ± 1.2 ^a,c^	76.3 ± 1.0 ^b,c^	<0.001
% body fat	21.8 ± 1.2 ^a,b^	25.2 ± 1.0 ^a,c^	29.1 ± 0.8 ^b,c^	<0.001
Visceral adipose tissue, cm^2^	14.8 ± 1.2 ^a,b^	17.9 ± 1.2 ^a^	20.2 ± 0.9 ^b^	0.002
Subcutaneous adipose tissue, cm^2^	50.32 ± 6.7 ^a,b^	74.3 ± 6.6 ^a,c^	97.7 ± 5.3 ^b.c^	<0.001
*Lifestyle factors*				
TV hours/day	2.7 ± 0.2	2.8 ± 0.2	3.1 ± 0.2	0.23
> 2 hours of TV/day, %	50.0±4.7 ^b^	60.0 ± 5.7	67.2 ± 0.4 ^b^	0.02
Computer or video, hours/day	2.1 ± 0.2	2.2 ± 0.2	2.2 ± 0.2	0.93
Total screen time, hours/day*	4.8 ± 0.3	5.0 ± 0.3	5.3 ± 0.30	0.50
> 2 hours of total screen time/day, %*	70.4 ± 3.8 ^a, b^	82.3 ± 3.8 ^a^	85.1 ± 3.0 ^b^	0.009
Physical activity sessions/week	8.6 ± 0.7	8.6 ± 0.7	8.7 ± 0.6	0.98

*Total screen time: n=369 for offspring.

a: p<0.05 between normal weight and overweight;

b: p<0.05 between normal weight and obese;

c: p<0.05 between overweight and obese.

Examining adiposity measures stratified across tertiles of total screen time for both offspring and parents showed that BMI, waist circumference, % body fat, and visceral fat were greater with increasing number of hours spent watching TV, using the computer, or playing video games (data not shown).

### Comparison between parents when they were children (parent_child_) and their offspring

**Figure 2:**
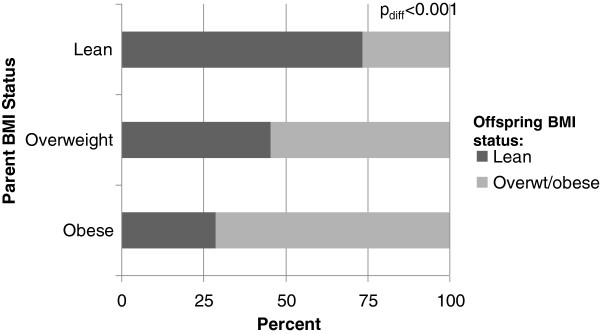
Distribution of normal weight, overweight, and obese children according to parental child BMI status (as children).

The BMI of the parents when they were the same age as their offspring was significantly lower than the BMI of their offspring (Table 3), with a significantly lower prevalence of obesity. However, correlations between the parents_child_ and their offspring were significant for BMI and BMI percentiles, even after adjustment for age, sex, and race. As with parent_adult_ BMI status, the distribution of BMI categories in offspring was significantly associated with parent_child_ BMI status (Figure 2). When examining BMI of offspring across BMI categories of their parents_child_, mean BMI was significantly higher in the offspring of parents who were overweight or obese in childhood than in offspring whose parents were normal weight as children (data not shown). Mean hours of TV watched was similar between parents_child_ and offspring. Therefore, it is not surprising that mean hours of TV watched by the parents_child_ were significantly correlated with the number of hours of TV currently watched by their offspring (Table 3; p<0.001). Finally, we found that including both parental (child and adult) BMI measures explained more of the variance of offspring BMI than either measure alone (parent_child_+parent_adult_ BMI=52%; parent_adult_ BMI=49%; parent_child_=50% (52% vs 49% or 50%; both p_diff_<0.001).

**Table 3:** Mean ± SE or frequency (%) of measures of adiposity for parents as children and their offspring adjusted for age, sex, and race, (n=382)

**Characteristics**	**Parent as child n=382**^**1**^	**Offspring n=382**	**p-value**	**Pearson correlation**^**2**^
Age, years	12.6 ± 0.2	12.3 ± 0.2	0.25	--
*Clinical characteristics*				
Height, cm	148.0 ± 0.5	151.3 ± 0.5	<0.001	0.47**
Weight, kg	46.8 ± 0.8	52.3 ± 0.7	<0.001	0.49**
BMI, Kg/m^2^	20.2 ± 0.2	21.5 ± 0.2	<0.001	0.44**
BMI percentiles, %	61.0 ± 1.6	64.1 ± 1.5	0.16	0.37**
BMI status, %				
Normal weight	71.8	64.6	0.03	--
Overweight	17.8	16.8	0.72	
Obese	10.4	18.6	<0.001	
*Lifestyle factors*				
TV hours watched ^3^	2.9 ± 1.9	2.9 ± 2.0	0.80	0.62**
> 2 hours of TV, %	59.4 ± 49.1	60.5 ± 48.9	0.39	

*NA* not available.

^1^ Some parents (n=234) had more than one child enrolled in this study and therefore, matched to each of their children (n=382).

^2^ Pearson partial correlations (adjusted for age, sex, and race) for measures of adiposity between parents in childhood and their offspring.

^3^ TV for the parent as a child was obtained at age 7, while TV for offspring was obtained at average age 12 years.

** p<0.001.

## Discussion

The present study shows that adiposity and hours of TV watched by offspring were significantly correlated with their parent’s adiposity and TV watching, both when the parents were children and at their current adult age. Consistent with well recognized secular trends, it was not surprising to observe greater prevalence of overweight and obesity among offspring than among the parents_child_. According to national surveys, the prevalence of obesity among children and adolescents ages 12–19 more than tripled from 5% in 1976–80 to 18.1% in 2007–08 [21], while the prevalence of obesity in adults doubled during this same time period from 15% in 1976–80 to 34.3% in 2007–08 [22]. It is unlikely that heredity is totally responsible for this substantial change over a short time period [23], thus, supporting the contribution of environmental factors in the development of obesity [24].

There were significant correlations between offspring BMI and both BMI of parents_child_ and parents_adult_. Although, both parental measures (as a child or adult) were slightly more predictive of offspring adiposity, each of childhood or adult parental BMI measure explained about 50% of the variance in offspring BMI. Genetic studies have shown heritability for BMI to be about 40-50% [23]. A significant correlation between offspring and their parents_adults_ was also observed for % body fat, which has a heritability estimate less than BMI [8,23]. As expected, offspring of parents who were obese both during childhood and as an adult have greater BMI than children of normal weight parents. Although there is a well-known strong tracking effect for BMI from childhood to adulthood [2,25,26], this study now shows an equally strong familial effect demonstrated by the relation of BMI and central adiposity status in parents to overweight/obesity in the next generations. A significant relation between self-reported CVD risk factors, including BMI, in parents and increased levels of CVD risk factors in their children was observed in the Bogalusa Heart Study [4,27]. Although prior studies have shown the parental influence on child obesity [2,3,5], the present study extends those results by being able to provide data from actual BMI measurements in parents when they were children as well as adults.

Although overall adiposity, BMI and % body fat, are positively related to chronic disease, measurement of waist circumference and visceral adipose tissue may be better indicators of degree of adiposity than BMI. Increased visceral adiposity is correlated with abdominal obesity, a predictor of CVD, and is associated with insulin resistance and CVD risk factors [28]. Based on family studies, heritability estimates for visceral adipose tissue range from 42% to 56% [8,9], heritability for waist circumference is 60% [29], and heritability estimates for subcutaneous fat range from 30-50% [8,30]. The findings in this study are consistent with genetic studies demonstrating greater waist circumference and visceral adipose tissue among offspring of abdominally obese parents compared to offspring of normal weight parents.

This study also found that screen time was significantly and positively correlated between parents both at their current age and when they were children and their offspring. The finding that screen time was higher in offspring than their parents in each of the BMI categories may help explain the greater degree of adiposity among offspring than their parents when they were at a similar age. Greater BMI in both parents and their offspring was associated with more hours of total screen time.

Family environment has been shown to influence children’s TV viewing habits and level of activity [31,32], supporting the present findings that TV and screen time behavior is qualitatively similar between parents and children [13]. Consistent with prior studies, a greater proportion of offspring with overweight or obese parents reported 2 or more hours of TV or total screen time [13,14]. In cross-sectional studies of children more hours of TV watching or screen use (computers, video games) were associated with overweight and obesity [13,33,34]. Preschool children watching > 2 hours per day of TV or playing video games were 34% more likely to be overweight or at risk for overweight than those watching less than 2 hours daily [33]. Adolescents engaged in two or more hours of screen time (TV, computer, or video games) have over two times the odds of being overweight or obese than those reporting fewer hours [13]. An important observation in the present study is that a majority of offspring and their parents, both when they were children and currently as adults reported watching 2 or more hours of TV and total screen time daily, which is greater than screen time recommendations by the American Academy of Pediatrics [35]. Because sedentary behavior appears to track from parents to their young offspring, strategies to reduce levels of childhood overweight/obesity need to effectively change parental as well as childhood screen time [14,25,32,36].

Few reports have been published about parental influence on their offspring’s physical activities. The level of physical activity in the present study was greater in offspring than their adult parents, but in contrast to total screen time, there was not a significant correlation between parents and offspring. A recent longitudinal study of factors influencing eating and physical activity in families reported a positive association between encouragement from parents to be active and increased physical activity among their offspring [32]. However, results from observational studies are inconsistent, with some reporting positive relations between parental influence on their offspring’s physical activity habits and others reporting no effects of parental influence [32,37,38]. In the present study offspring self-reported significantly more physical activity sessions per week than their adult parents, but there was no relation between the number of offspring physical activity sessions and parent BMI categories. According to self-report information, 31 percent of U.S. adults participate in physical activity, either three times/week of vigorous physical activity lasting 20 minutes or more or five times per week of light-to-moderate physical activity lasting 30 minutes or more, while 40 percent of adults report no leisure-time physical activity [39]. However, self-reports appear to be woefully inadequate. When physical activity was measured by a device that detects movement, only about 3–5 percent of adults completed 30 minutes of moderate or greater intensity physical activity on 5 or more days per week [40]. Among high school students, 35 percent reported participation in at least 60 minutes of physical activity on 5 or more days of the week [41], but only 8 percent achieved this goal when measured by an activity device [40].

Limitations include that only one parent was enrolled in the study instead of both parents; however, most studies report similar influences by either parent on the BMI of daughters or sons [5,42]. There was no apparent correlation of usual physical activity between offspring and their parents, which may be due to under- or over-reporting of physical activities [43]. However, the physical activity questionnaire administered to parents as children was slightly different than the questionnaire administered to the offspring. TV watching was significantly correlated between offspring and their parents suggesting that self-reported hours of watching TV is moderately reliable [44] or that parental behavior shapes their child’s sedentary behavior, but that level of physical activity may be dictated by activity outside the home.

Strengths of this study include the use of an established cohort with long-term measures of parents from when they were children (age 7) to current adulthood (age 40 years) and measures on their young offspring; collection of data from parents as children and as adults and their offspring using the same standardized questionnaires, except for physical activity in parents as children; and procedures to measure anthropometrics, total body fat by DXA, and visceral adiposity as assessed by CT scan in both parents and offspring.

## Conclusions

Heredity may contribute from 25-55% of the risk for obesity [23], and is supported by the significant correlations between offspring and their parents when they were the same childhood age. However, the exponential increase in the prevalence of overweight and obesity over the past 25 years is likely due to factors other than genetics, since the genes of a population do not change that rapidly. The present study reporting parental/offspring correlations for both fatness and screen time suggest both a genetic and environmental influence on the relation of obesity between parents and their offspring during their early years and provides support for the initiation of family oriented interventions in an attempt to prevent obesity during childhood and adolescence and eventual adult CVD.

## Abbreviations

CVD: Cardiovascular disease; VAT: Visceral adipose tissue; SAT: Subcutaneous adipose tissue; TV: Television; Parentsadults: Parents as adults; BMI: Body mass index; Parentschild: Parents when they were children; DXA: Dual-energy x-ray absorptiometry; HU: Houndsfield units; SD: Standard deviation.

## Competing interests

The authors have no Competing interest to disclose.

## Author’ contributions

JS, DRJ, and ARS designed the study; JS, DRD, and LSC carried out the study; LMS, ARS, and JS wrote the paper, LMS and XZ analyzed the data. All authors were involved in reviewing and revising the paper, interpreting the data, and had final approval of the submitted and published versions.

## Pre-publication history

The pre-publication history for this paper can be accessed here:

http://www.biomedcentral.com/1471-2431/13/133/prepub
